# Reply to “A Thermodynamic assessment of the reported room-temperature chemical synthesis of C_2_”

**DOI:** 10.1038/s41467-021-21439-2

**Published:** 2021-02-23

**Authors:** Kazunori Miyamoto, Shodai Narita, Yui Masumoto, Takahiro Hashishin, Taisei Osawa, Mutsumi Kimura, Masahito Ochiai, Masanobu Uchiyama

**Affiliations:** 1 Graduate School of Pharmaceutical Sciences, The University of Tokyo, Tokyo, Japan; 2 Division of Chemistry and Materials, Faculty of Textile Science and Technology, Shinshu University, Ueda, Japan; 3 Research Initiative for Supra-Materials (RISM), Shinshu University, Ueda, Japan; 4 Graduate School of Pharmaceutical Sciences, University of Tokushima, Tokushima, Japan; 5 Cluster of Pioneering Research (CPR), Advanced Elements Chemistry Laboratory, RIKEN, Saitama, Japan

**Keywords:** Organic chemistry, Physical chemistry, Theoretical chemistry

**Replying to H. Rzepa.** *Nature Communications* 10.1038/s41467-021-21433-8 (2021)

We are writing in response to Rzepa’s theoretical analysis of the generation of C_2_, which cites our recent paper describing the first chemical synthesis of C_2_ (ref. ^1^). Rzepa suggests on the basis of several in silico approaches that the formation of free C_2_ from alkynyl-λ^3^-iodane and fluoride ion would be prohibitively endo-energetic. He proposes three possible explanations of the apparent discrepancy between these theoretical calculations and our experimental findings, of which one is that some species other than C_2_ is actually formed.

As our original paper was primarily experimental, describing room-temperature chemical synthesis of C_2_ and the first bottom-up chemical synthesis of nanocarbons from C_2_, we should like to respond to the latter point. We believe that the evidence presented in our paper for the generation of C_2_ itself is compelling^1^. In particular, the connected-flask, solvent-free experiment clearly supports the generation of free C_2_ gas, for the following reasons:

1. When ^13^*C*-labeled **1b**-^13^*C*_β_ was used, a 1:1 mixture of *O*- ethynyl galvinoxyl **15**-^13^*C*_α_ and **15**-^13^*C*_β_ was obtained (Fig. 1a).

**Fig. 1:**
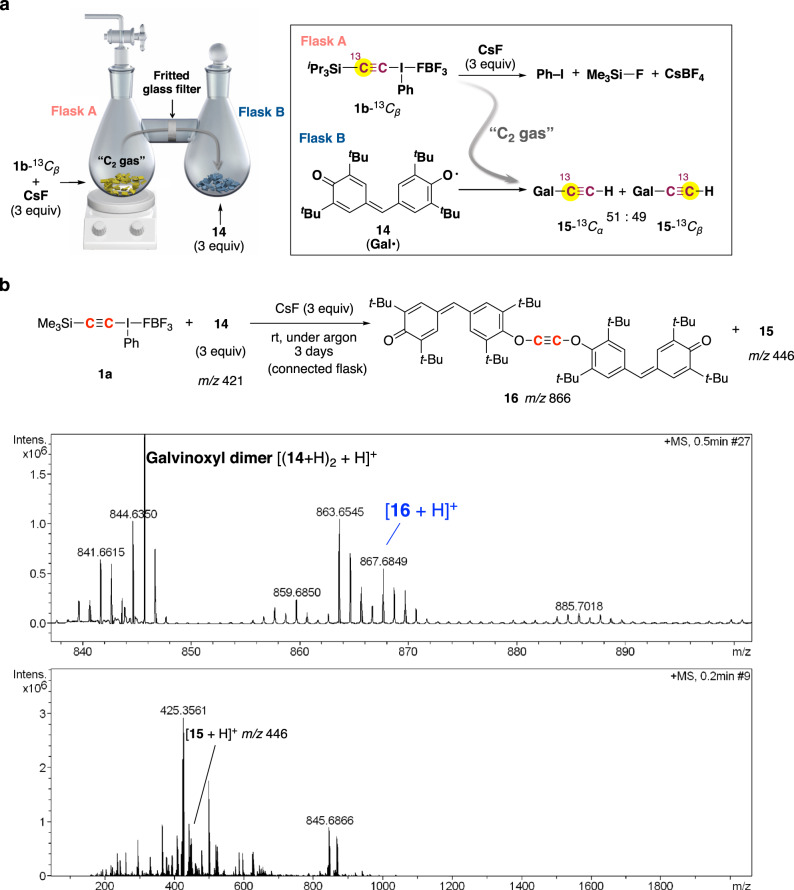
Connected-flask, solvent-free experiment. **a** ^13^C-labeling experiment. **b** APCI mass spectrum (positive ion mode) obtained from the contents in Flask B.
2. APCI mass spectrum of the contents in Flask B included the peak assignable to acetylene digalvinoxyl ether **16** (Fig. 1b).

It is more difficult to establish conclusively whether free C_2_ is generated in solution, but the following experimental facts are relevant:

3. The relative rate of hydrogen abstraction between CH_2_Cl_2_ and 9,10-dihydroanthracene (**12**) is calculated to be ca. 1:20 (per 1H).
4. Galvinoxyl radical **14** solely produced *O*-ethynyl galvinoxyl **15** (not **16**).
5. The amount of the mixture of **15**-^13^C_α_ and **15**-^13^C_β_ obtained by the reaction of **1b**-^13^C_β_ with **14** differed in solvents of different viscosities (*η*).

These results indicated that the radical character of the intermediate is much milder than that of the common unstabilized alkynyl radical, which is consistent with the presence of an interaction between the radicals in C_2_, in other words, a singlet biradical (charge-shift bonding) character, as suggested in a recent theoretical study^2^.

At present, we cannot explain the discrepancy between Rzepa’s theoretical evaluation and our experimental results. However, as discussed in our original paper, we believe that all our experimental observations can only be rationalized in terms of the generation of gaseous C_2_. Our findings have attracted great interest, and we anticipate that independent experimental findings will emerge in the near future. We ourselves are working on the direct observation of chemically generated C_2_ by means of Raman spectroscopy, ESR spectroscopy, and other methods, and we hope that this work will provide definitive experimental evidence for the bond length and electronic state of C_2_ (ref. ^3^ and references therein).

## Data Availability

The data that support the findings of this study are available from the authors on request.

## References

[1] Miyamoto K (2020). Room-temperature chemical synthesis of C_2_. Nat. Commun..

[2] Shaik S (2020). Charge-shift bonding: a new and unique form of bonding. Angew. Chem. Int. Ed. Engl..

[3] Shaik S, Danovich D, Braida B, Hiberty PC (2016). The quadruple bonding in C_2_ reproduces the properties of the molecule. Chem. Eur. J..

